# Fatal Case from the Inhalation of Chloroform

**Published:** 1854-03

**Authors:** 


*Fatal Case from the Inhalation of Chloroform.* By JAMES DUNSMURE, M. D., Surgeon to the Royal Infirmary of Edinburgh.

As I consider it proper that every death from the inhalation of chloroform should be recorded, and as the first fatal case in Edinburgh has lately occurred in the Royal Infirmary, in a patient under my care, upon whom I was going to operate, I take the earliest opportunity of communicating the particulars of it to my professional bretnren, and I do so the more readily as in some respects the manner of death differed from what has been observed in similar melancholy instances.

In a paper published by Mr. E. R. Bickersteth, of Liverpool, in the "Monthly Journal for September, 1853," on the mode of death from the inhalation of chloroform, he states, as his opinion, from experiments he has performed on animals, and from the observation of cases in which chloroform nearly proved fatal when inhaled for the purpose of producing anæsthesia, that death begins at the lungs, and that the cessation of the heart's action is secondary. In other words, that the breathing stops before the heart has ceased to beat. But in the case which I am now about to relate, the pulse seemed to stop before the breathing. I cannot assert this positively from my own observation, as from the position I occupied when about to commence the operation, I was unable to see the respiratory movement of the chest; but from the evidence of my colleague, Mr. Spens, and Dr. Struthers, my house-surgeon, who watched the pulse, and could and did observe the breathing, there can be little doubt that the weakness of the pulse was the first cause of alarm, and that its complete cessation was noticed before the breathing stopped.

The history of the case is as follows:

John Mitchell, æt. 43, a tobacconist, of intemperate habits, was admitted into Ward No. 22, on the 20th of June last, for retention of urine, having been transferred to the Surgical Hospital, on account of urinary symptoms, from one of the medical wards where he had been under treatment for some time for an attack of pneumonia. No. 1 catheter, with some difficulty, was passed through the stricture into the bladder, and tied in, with the intention of allowing it to remain for twenty-four hours; but from the pain, severe rigors, and general febrile disturbance it occasioned, the instrument was withdrawn in twelve hours. He stated that he had suffered from stricture for eighteen years, that no instrument had ever been passed into his bladder, and that for many months before his admission into the Infirmary, his urine had come from him in drops, and during sleep was voided involuntarily. Every attempt to pass the catheter produced a shivering fit, and as his health was impaired from his previous habits, and likewise weakened by the attack of pneumonia, from which he had hardly recovered, I determined, as he could now micturate in a small stream, to discontinue, for a time, the use of the catheter, and merely endeavor to improve his general condition. He soon began to regain strength, and it was again resolved to pass instruments through his stricture, and to divide it by an incision made in the perineum as soon as No. 2 staff could be introduced. From the unyielding nature of the stricture, and from my unwillingness to persevere too long at each attempt to pass No. 2, from fear of producing rigors, which still threatened to come on after every introduction of No. 1, but which were kept off by warm fomentations to the perineum, I did not succeed in getting No. 2 staff passed until Wednesday the 28th of September. The nature of the proposed operation having been explained to the patient, and his consent obtained to its performance, he was removed to the small operating theater of the hospital. I was assisted by my colleague, Mr. Spens, my clerks and dressers. According to the practice in our Infirmary, the chloroform was given by Dr. Struthers, my house-surgeon; and I may here mention that this gentleman, before entering on his duties in the Surgical Hospital, had acted for eighteen months as resident and non-resident clerk in the obstetrical ward, under the charge of Dr. Simpson, where he had been constantly in the habit of administering it. While the patient was inhaling the drug, he struggled considerably, and became a good deal congested in the face and head. He seemed to take a slight convulsion, like an epileptic fit, and such as I have seen on several occasions in the people who have led an intemperate life. During the convulsion, the handkerchief, containing the chloroform, was removed to some distance from the face. In a short time the inhalation took effect, and he began to snore, and although still violent, the chloroform was removed from the face entirely, and the handkerchief placed under the pillow. As soon as the patient became more quiet, he was pulled down on the table, and placed in the proper position for the operation. I then shaved the perineum, and was just going to make my first incision, when one of the assistants said that his pulse was becoming weak. The posterior tibial, Mr. Spence then remarked, was good, but in a second or two after, both gentlemen exclaimed that the pulse was gone. I rushed from my seat to the patient's head, and found that his breathing had ceased. Those present who had an opportunity of observing the respiration, which I had not, owing to the stool on which I sat being low, positively assert that the breathing did not cease before the pulse. The face was much congested, the jaws were firmly closed, and the pupils were dilated. I immediately forced open the lower jaw by means of the handle of a staff, and with catch forceps pulled out the tongue. Artificial respiration was had recourse to, and in a few moments he made a long inspiration. This was soon followed by a second, by a third at a longer interval, by a fourth at a still longer period, and then by a fifth, when all attempts at natural breathing ceased. No pulsation could be felt in the radial arteries. The chest was noticed to be much contracted, to have apparently lost its elasticity, and not to expand when the ribs were forcibly compressed during the artificial respiration. I had previously sent for a galvanic apparatus, which was in the flat below, and it arrived almost immediately after the patient had made the fifth inspiration. When the tongue was pulled out, and before the first breath was taken, I was on the point of opening the trachea, but this proceeding was then abandoned; it was now, however, had recourse to, in order to carry on artificial respiration more certainly; the external jugular was also opened, and about a couple of ounces of blood flowed. By the time the tracheotomy tube was inserted, the galvanic apparatus was in working condition, and it was applied on each side of the diaphragm. It acted remarkably well; at each application of the sponges, the muscle descended as if the patient was in life; air passed through the tube in the trachea, and for some time I was in great hopes that the man was to be saved; but the muscle gradually lost its contractility, and although the galvanism was kept up for an hour, it was evident that all our efforts were in vain—that life was extinct.

The post-mortem examination was made the following day at one o'clock, rather more than twenty-four hours after the patient's death, and I give the report of it drawn up by Dr. Gairdner, the pathologist to the Infirmary:

"John Mitchell, æt. 43, died 28th September. A very robust man; height five feet eight inches; diameter (lateral) of base of thorax, ten and a half inches.

"*External appearances.*—Considerable lividity of face and neck, and more than usual congestion of depending parts. Considerable amount of fat, but more in omentum and around viscera of abdomen than in external parietes.

"*Chest.*—Right pleura presents a few slight adhesions near the middle; left pleura free. No fluid in either pleural cavity. *Pericardium* contained about half an ounce of serum, and presented a few opaque patches on its surface. *Both* sides of heart contained blood, the right side rather more than the left. Blood more than usually fluid. External fat of heart considerable, about three lines on some parts of right ventricle. Muscular t
iissue of heart generally flabby, and rather pale, but not distinctly disorganed to the naked eye. Valves perfectly healthy. Aorta presented very faint traces of atheroma. A few traces of atrophy of right lung toward its apex and anterior edge, but very limited. In all other respects lungs free from disease, but somewhat congested.

" *Spleen* soft but not diffluent.

" *Liver* congested, but otherwise normal.

" *Kidneys* congested, but otherwise healthy.

" *Brain.*—The subarachnoid fluid presents considerable milky opacity, and is of moderate quantity. Moderate congestion of the meninges generally. About half an ounce of fluid in the ventricles. *Substance* of brain healthy. *Arteries* at base perfectly free from atheroma. *Air passages.*—Glottis perfectly patent. Mucous membrane of larynx and trachea slightly congested.

" *Microscopic examination* showed the *fibres of the heart* to be nearly normal, though scarcely so distinctly striated as in some cases. The *minute vessels* of the brain and pia mater presented at some points a few clustering granules, but to no great extent."

On a view of this most melancholy case, the questions that naturally suggest themselves are these:

1st. Was the chloroform bad?

2d. Was there any thing faulty in the way the chloroform was administered?

3d. Was there any peculiarity in the patient's constitution?

The first query is easily disposed of. The chloroform was procured from Messrs. Duncan and Flockhart, and is the same that I and my colleagues are constantly in the habit of using, both in hospital and in private practice, without ever having seen any cause for doubting its purity, so that I have not the slightest reason for thinking that the fatal event was owing to the impurity of the drug.

2d. The mode of administration. The chloroform was put upon a handkerchief, which was held at the distance of a few inches from the mouth, to enable the patient, during the inhalation, to inspire air along with the chloroform vapor, and I confess that I know no better method of administering it. The quantity used was about an ounce, which, considering the man's previous habits, was not a large dose. I have frequently seen administered, and given myself, a much larger quantity before a patient of like habits could be brought under its influence. I cannot, in fact, attach any blame to the mode of administration.

3d. Was there any peculiarity in the patient's constitution? There was nothing, so far as I could judge, to contra-indicate the use of chloroform. He had twice before inhaled it by my directions. On each of these occasions, he had a great deal of struggling, with congestion of the face and head; and it required as large a dose to make him insensible as on the day when the fatal event occurred. No bad effects followed the first and second inhalations, and I saw nothing to prevent me having recourse to it a third time. That it was a pure death from chloroform, no one can deny; but I have the consolation of feeling convinced that the ordinary precautions were had recourse to in the administration of the anæsthetic, and that there was nothing in our past experience of chloroform, or in the patient's history, to contra-indicate its employment. As far as I can calculate, several minutes must have elapsed from the time the handkerchief was removed from the face, until the pulse was observed to become weak. He had to be pulled down to the end of the table, his limbs had to be bent, which, from his struggling, and complete relaxation not having occurred, took some time to do; his perineum had to be shaved, all which operations, I am confident, occupied four or five minutes. During this time he was observed to be breathing stertorously, and yet the influence of the chloroform was accumulating, as is evident from respiration not having become re-established, although five inspirations were made after the tongue was pulled out of the mouth. In several instances where I have seen chloroform very nearly prove fatal, the respiration became gradually restored after an inspiration had once been made; in this case, hawever, no such fortunate occurrence took place.

As mentioned in the account of the post-mortem examination, the glottis was found quite patent, thus showing that the opening of the trachea could not have been attended with any benefit. The patient seemed to take an epileptic fit, and the operation was had recourse to in case the larynx was partially obstructed with mucus; but if artificial respiration, by compressing the ribs and blowing into the mouth, when the tongue is held out, does not restore the breathing, I do not think that the chance of recovery is increased by tracheotomy. Great benefit, in cases similar to this, is likely to be derived from galvanism, and I feel almost persuaded that if, in this instance, it could have been had recourse to a few minutes earlier, the patient's life might have been saved. I would suggest that in a public hospital where there is always a galvanic apparatus, that it should be near at hand when chloroform is being administered.

I would recommend, also, when there is much struggling, and when apparently there is a tendency to convulsion, that the handkerchief be removed for a few seconds from the mouth, or, at all events, held at a greater distance from it than is generally done. The patient, no doubt, will be longer in coming under the influence of the chloroform, but its vapor will be more thoroughly mixed with atmospheric air before being inhaled. This caution, in my opinion, is particularly necessary in people who have led an intemperate life, as it is in them that the greatest struggling and tendency to convulsion are observed. They do not breathe freely, and if the handkerchief, as is too often the case, is forcibly applied over the mouth, there is great risk of half choking them, or, at all events, of preventing a sufficient quantity of air passing into the lungs, and thus producing the poisonous effects of the anæsthetic. That fatal cases, from some peculiarity of constitution, will occur, from time to time, I do not doubt, and I only trust that the knowledge that such cases may happen, will be the means of making those who use chloroform careful to watch for the first alarming symptoms either in the breathing or in the pulse.

EDINBURGH, 57 Queen street, 5th Oct., 1853.—*Monthly Journal of Med. Science.*

